# Revision for cage migration after transforaminal/posterior lumbar interbody fusion: how to perform revision surgery?

**DOI:** 10.1186/s12893-022-01620-0

**Published:** 2022-05-11

**Authors:** Masato Tanaka, Zhang Wei, Akihiro Kanamaru, Shin Masuda, Yoshihiro Fujiwara, Koji Uotani, Shinya Arataki, Taro Yamauchi

**Affiliations:** 1 Department of Orthopaedic Surgery, Okayama Rosai Hospital, 1-10-25 Chikkomidorimachi Minami Ward Okayama, Okayama, 702-8055 Japan; 2 Department of Orthopaedic Surgery, Inner Mongolia Medical University Affiliated Hospital, Hohhot, 010050 Inner Mongolia Autonomous Region China

**Keywords:** Lumbar interbody fusion, Cage protrusion, Revision surgery, C-arm free, Pseudoarthrosis

## Abstract

**Background:**

Symptomatic pseudarthrosis and cage migration/protrusion are difficult complications of transforaminal or posterior lumbar interbody fusion (TLIF/PLIF). If the patient experiences severe radicular symptoms due to cage protrusion, removal of the migrated cage is necessary. However, this procedure is sometimes very challenging because epidural adhesions and fibrous union can be present between the cage and vertebrae. We describe a novel classification and technique utilizing a navigated osteotome and the oblique lumbar interbody fusion at L5/S1 (OLIF51) technique to address this problem.

**Methods:**

This retrospective study investigated consecutive patients with degenerative lumbar diseases who underwent TLIF/PLIF. Symptomatic cage migration was evaluated by direct examination, radiography, and/or computed tomography (CT) at 1, 3, 6, 12, and 24 months of follow-up. Cage migration/protrusion was defined as symptomatic cage protrusion > 5 mm from the posterior border of the over and underlying vertebral body compared with initial CT. We evaluated patient characteristics including body mass index, smoking history, fusion level, and cage type. A total of 113 patients underwent PLIF/TLIF (PLIF n = 30, TLIF n = 83), with a mean age of 71.1 years (range, 28–87 years). Mean duration of follow-up was 25 months (range, 12–47 months).

**Results:**

Cage migration was identified in 5 of 113 patients (4.4%). All cases of symptomatic cage migration involved the L5/S1 level and the TLIF procedure. Risk factors for cage protrusion were age (younger), sex (male), and level (L5/S1). The mean duration to onset of cage protrusion was 3.2 months (range, 2–6 months). We applied a new classification for cage protrusion: type 1, only low back pain without new radicular symptoms; type 2, low back pain with minor radicular symptoms; or type 3, cauda equina syndrome and/or severe radicular symptoms. According to our classification, one patient was in type 1, three patients were in type 2, and one patient was in type 3. For all cases of cage migration, revision surgery was performed using a navigated high-speed burr and osteotome, and the patient in group 1 underwent additional PLIF without removal of the protruding cage. Three revision surgeries (group 2) involved removal of the protruding cage and PLIF, and one revision surgery (group 3) involved anterior removal of the cage and OLIF51 fusion.

**Conclusions:**

The navigated high-speed burr, navigated osteotome, and OLIF51 technique appear very useful for removing a cage with fibrous union from the disc in patients with pseudarthrosis. This new technique makes revision surgery after cage migration much safer, and more effective. This technique also reduces the need for fluoroscopy.

## Introduction

Spinal instability due to degenerative, traumatic, infectious and neoplastic diseases may require fusion. Minimally invasive posterior and transforaminal lumbar interbody fusion (MI-PLIF/TLIF) have become established procedures for this purpose [1–3]. However, symptomatic pseudarthrosis and cage migration/protrusion are among the most difficult complications for MI-PLIF/TLIF. This cage migration may cause direct compression of the nerve root and/or cauda equina, pseudoarthrosis, and instrument failure. Several reports have emphasized that cage positioning in the disc space is an important factor in cage migration [4, 5]. If the patient develops severe radicular symptoms due to cage migration, removal of the cage becomes necessary. However, such removal is sometimes challenging if epidural adhesion or fibrous union has developed between the cage and vertebrae. We describe herein a new classification of cage protrusion after MI-PLIF/TLIF and a novel technique utilizing a C-arm-free navigated osteotome and the oblique lumbar interbody fusion at L5/S1 (OLIF51) technique to address these complications.

## Methods

**Fig. 1:**
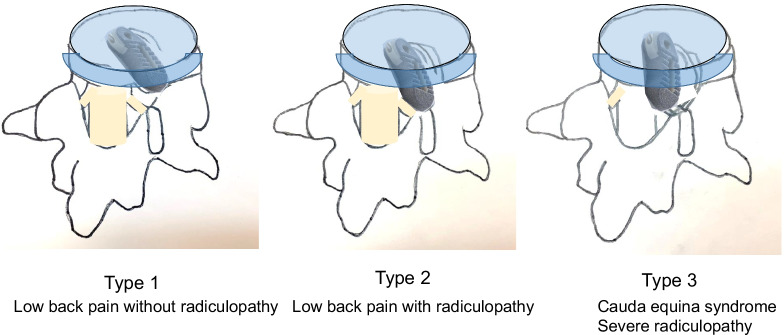
Type1: low back pain without radiculopathy. Type 2: low back pain with radiculopathy. Type 3: cauda equina syndrome severe radiculopathy

This retrospective study was approved by the institutional review board of our hospital (approval no. 283), in accordance with the research guidelines for humans. The inclusion criteria for patients were: MI-PLIF/TLIF in our hospital between July 2017 and December 2020; and follow-up for > 1 year. Exclusion criteria were: severe osteoporosis; rheumatoid arthritis; or destructive spondyloarthropathy. A total of 113 consecutive patients were enrolled in this study. We use our new classification of the cage protrusion; type 1; only low back pain without new radicular symptom, type 2; low back pain with slight radicular symptom, type 3; cauda equine syndrome and/or severe radicular symptom (Fig. 1). For each patient, direct examination, postoperative lumbar radiograms and/or computed tomography (CT) were obtained at 1, 3, 6, 12, and 24 months of follow-up. Cage migration was defined as symptomatic posterior cage protrusion > 5 mm from the posterior border of the over and underlying vertebral body compared with initial CT. We evaluated patients’ characteristics such as body mass index, smoking history, fusion level, and cage type.

We applied a new classification for cage protrusion: type 1, only low back pain without new radicular symptoms; type 2, low back pain with minor radicular symptoms; or type 3, cauda equina syndrome and/or severe radicular symptoms (Fig. 1).

## Results

### The patients’ demographic and surgical results

The patients’ demographic and surgical results were summarized in Table 1. Total 113 patients underwent MI-PLIF/TLIF; MI-PLIF 30, MI-TLIF 83, average 71.1 ± 9.7 years old (men 32, women 81, 28–87 years old). The average follows up period was 25.3 ± 5.3 months (12–35 months). Out of 113 patients, five cases of cage migration were identified, the incidence was 4.4%. The average of the cage protrusion patients was 56.6 years old, one woman and four men. The average on set of cage protrusion was 3.2 months (2–6 months). According to our classification, there were one patient in group 1, three patients in group 2, and one patient in group 3. The protrusion level was all L5/S1 level and this is statically significant (p = 0.021). Risk factors of cage migration were age (younger), gender (male), fusion level (L5/S1).

**Table 1:** Patient demographics

	Cage protrusion (−)	Cage protrusion (+)	*P* value
Patients	108 (95.6%)	5 (4.4%)	
Age	72.2 ± 9.9	56.6 ± 7.6	0.004**
BMI	22.7 ± 2.7	23.9 ± 1.3	0.085
Smoking history	33 (30.6%)	3 (60.0%)	0.167
Male/female	28/80(25.9%)	4/1 (80.0%)	0.006**
L5/S (%)	51/57 (44.4%)	5/0 (100%)	0.021*
MI-TLIF/PLIF	78/30(72.2%)	5/0(100%)	0.168

*P < 0.05, **P < 0.01

### Case presentation

#### Case 1: a 52-year-old man after left L5/S1 TLIF (Type 1 protrusion)

**Fig. 2:**
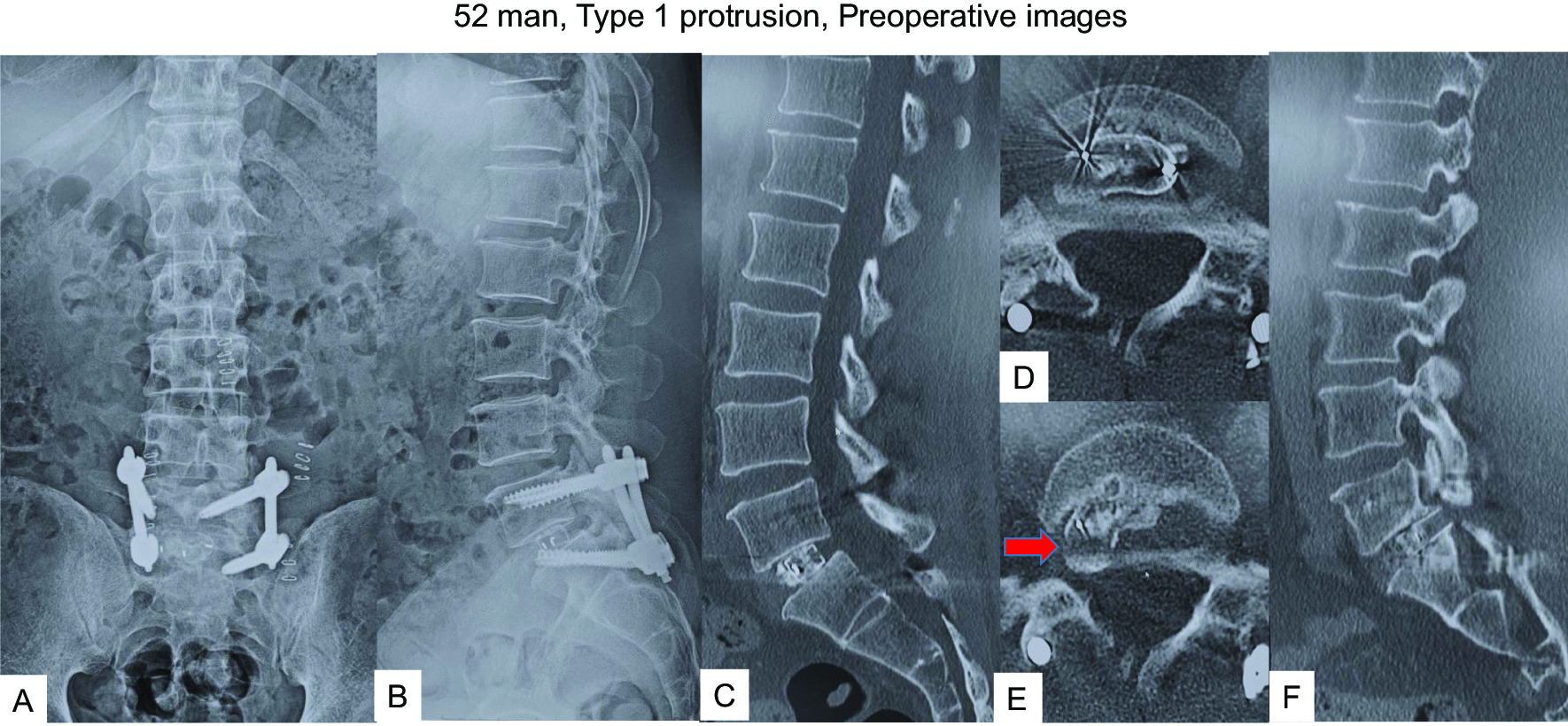
52 man, Type 1 protrusion, images before revision surgery **A** Postoperative anteroposterior radiogram, **B** Postoperative lateral radiogram, **C** Sagittal reconstruction CT at one month follow-up, **D** Axial CT at L5/S1 at one month follow-up, **E** Axial CT at L5/S1 at one year follow-up, **F** Sagittal reconstruction CT at one year follow-up. Red arrow shows a protruded cage

**Fig. 3:**
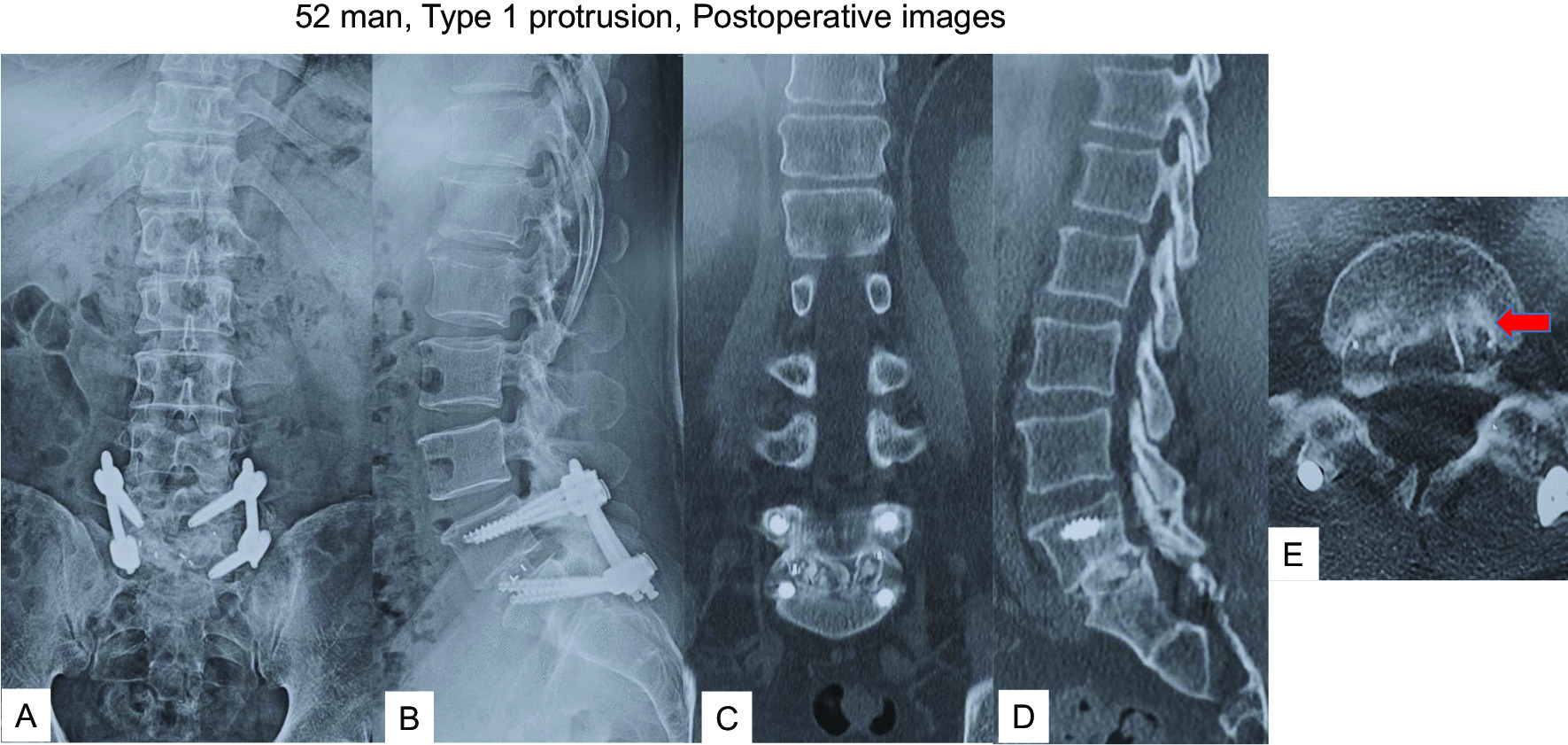
52 man, Type 1 protrusion, postoperative images **A** Final anteroposterior radiogram, **B** Final lateral radiogram, **C** Final coronal reconstruction CT, **D** Final sagittal reconstruction CT, **E** Final axial CT at L5/S1 Red arrow shows inserted new cage

The patient had undergone left L5/S1 MI-TLIF due to low back pain and left sciatica (Fig. 2A–D). From 6 months postoperatively, the patient complained of gradual recurrence of low back pain. Imaging at the 1-year follow-up showed posterior protrusion of the cage with no apparent bony fusion (pseudoarthrosis) (Fig. 2E, F). The patient complained of low back pain alone, with no radicular pain, and imaging indicated slight cage protrusion (Fig. 2E, arrow), so we decided to perform revision MI-TLIF from the contralateral side without cage removal (Fig. 3A, B). After this surgery, symptoms resolved and solid fusion had been obtained at the 1 year follow-up after revision surgery (Fig. 3C–E).

#### Case 2: a-66-year-old man after left L5/S1 MI-TLIF (Type 2 protrusion)

**Fig. 4:**
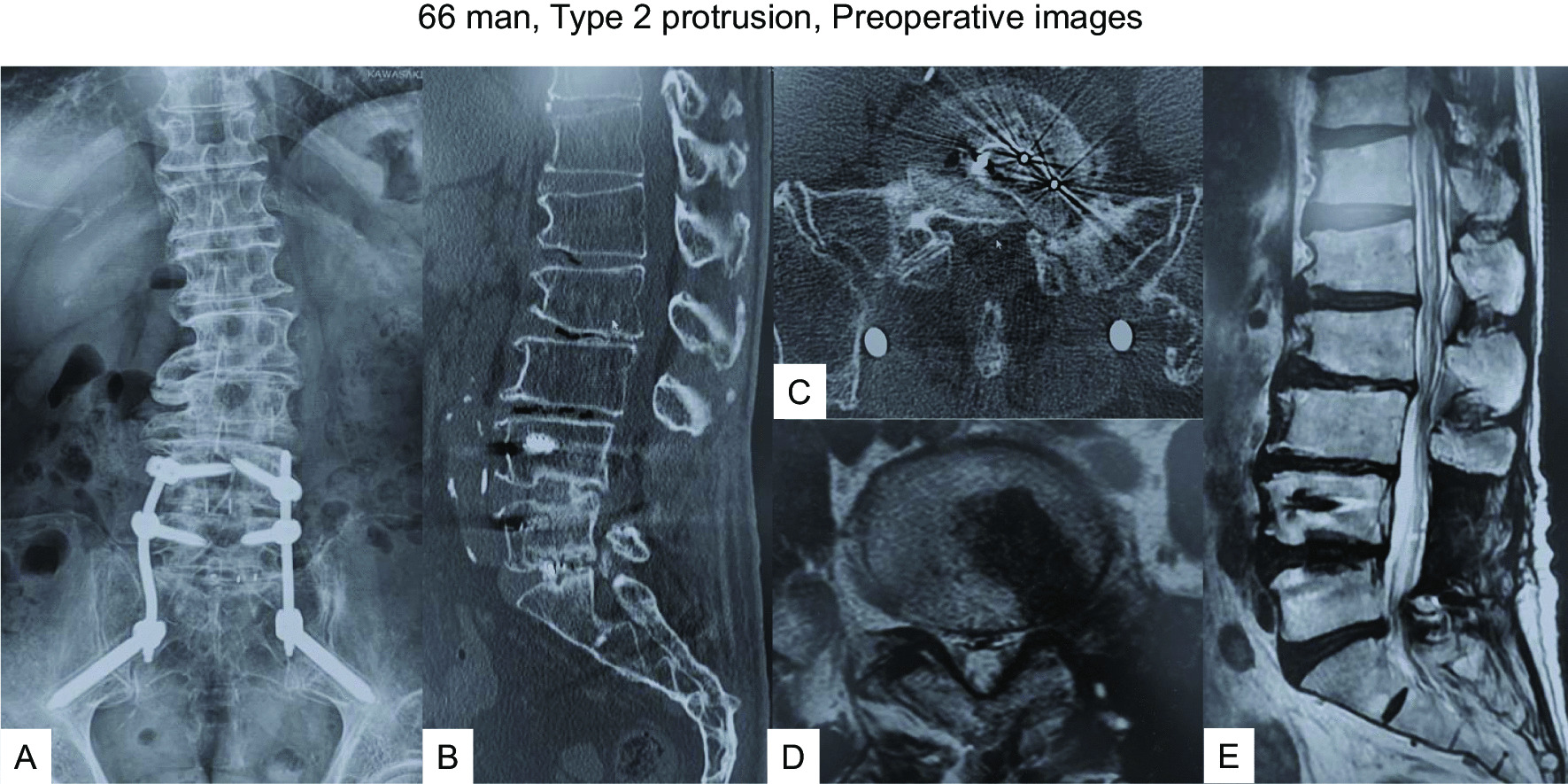
66 man, Type 2 protrusion, preoperative images **A**–**E**: Images at 5 months follow-up, **A** Anteroposterior radiogram, **B** Sagittal reconstruction CT, **C** Axial CT at L5/S1, **D** Axial T2-weighted MR imaging at L5/S1, **E** Sagittal T2-weighted MR imaging

**Fig. 5:**
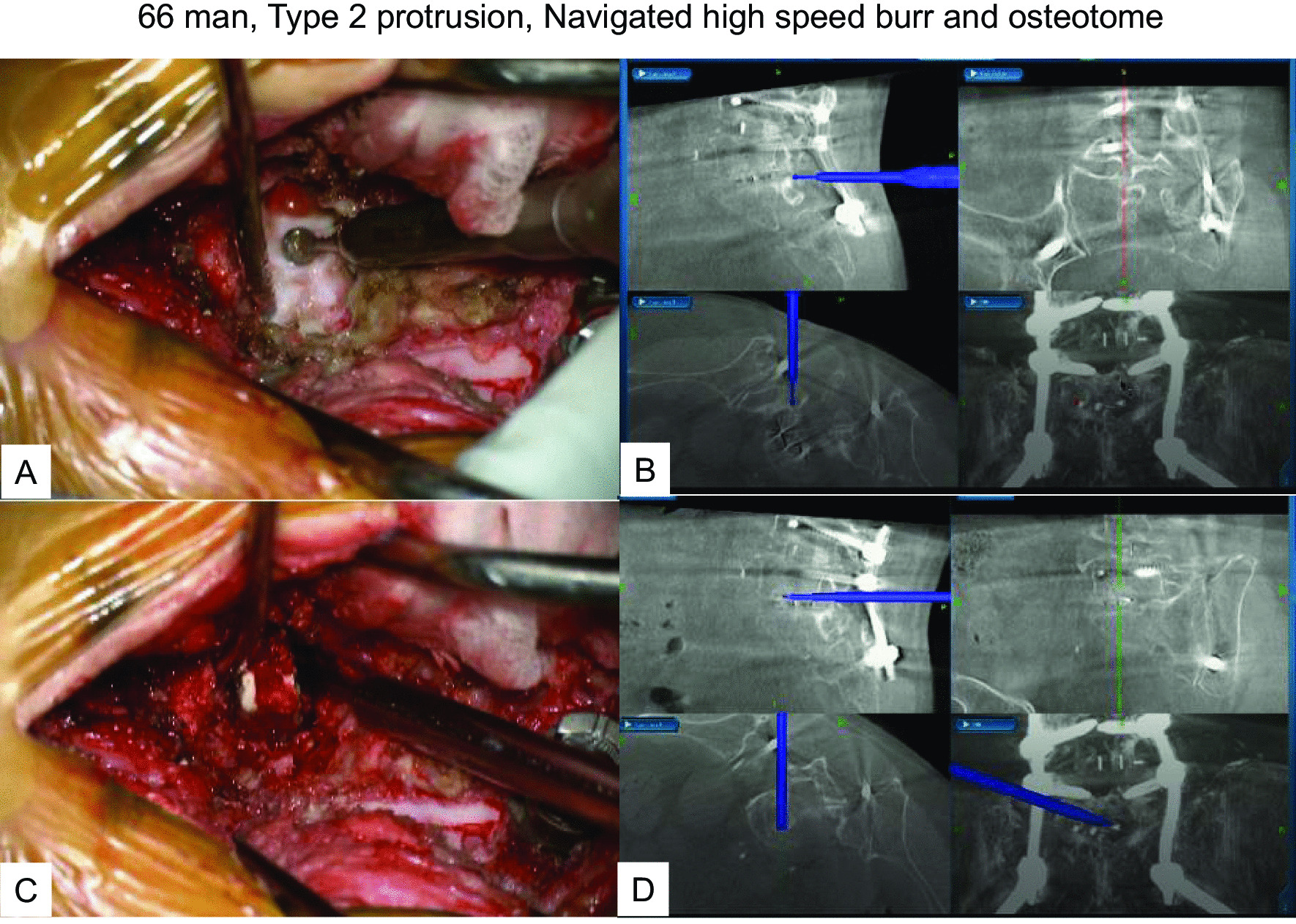
66 man, Type 2 protrusion, intraoperative and navigation images **A** Intraoperative image of a navigated high speed burr, **B** Navigation monitor of a navigated high speed burr, **C** Intraoperative image of a navigated osteotome, **D** Navigation monitor of a navigated osteotome

**Fig. 6:**
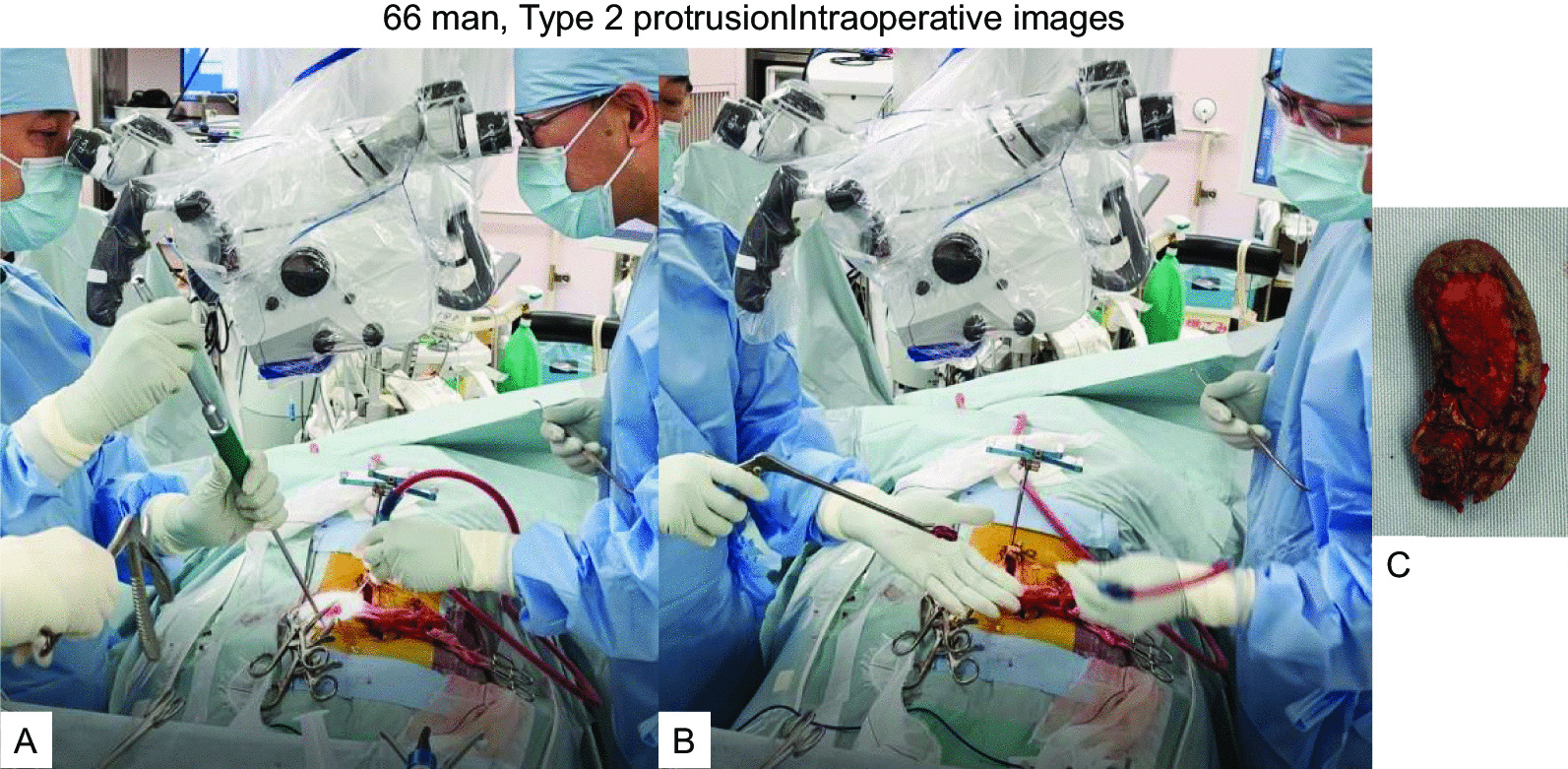
66 man, Type 2 protrusion intraoperative images **A** A cage remover, **B** The cage was removed by Kerrison rongeur, **C** The removed cage

**Fig. 7:**
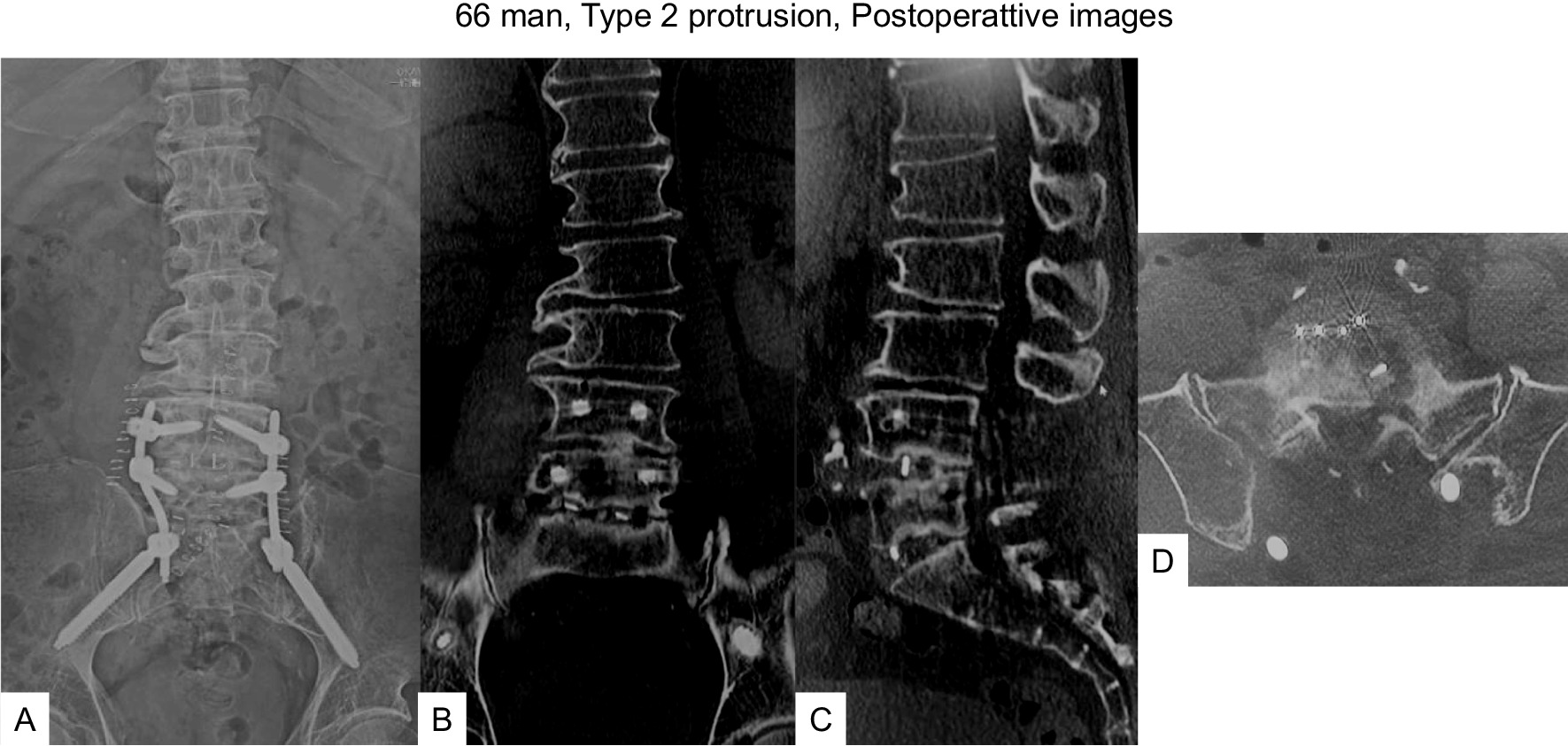
66 man, type 2 protrusion, postoperative images **A** Final anteroposterior radiogram, **B** Final coronal reconstruction CT, **C** Final sagittal reconstruction CT, **D** Final axial CT at L5/S1

The patient had undergone MI-TLIF at the L4/5 level in our hospital 15 months earlier due to left sciatica. Symptoms disappeared after the surgery, but from 5 months postoperatively, he started complaining of recurrent low back pain and left sciatica. Preoperative images indicated the cage at L5/S1 protruding into the spinal canal and compressing the left L5 nerve root (Fig. 4). Given the cage position, we decided to remove the cage from posteriorly under navigational guidance and neuromonitoring. The protruded cage had infiltrated the disc space, so we utilized a navigated high-speed burr (Fig. 5A, B) and navigated osteotomy (Fig. 5C, D) to release and remove the cage (Fig. 6). After cage removal, we performed L5/S1 MI-PLIF (Fig. 7).

#### Case 3: a 45-year-old woman, post left L5/S1 TLIF (Type 3 protrusion)

**Fig. 8:**
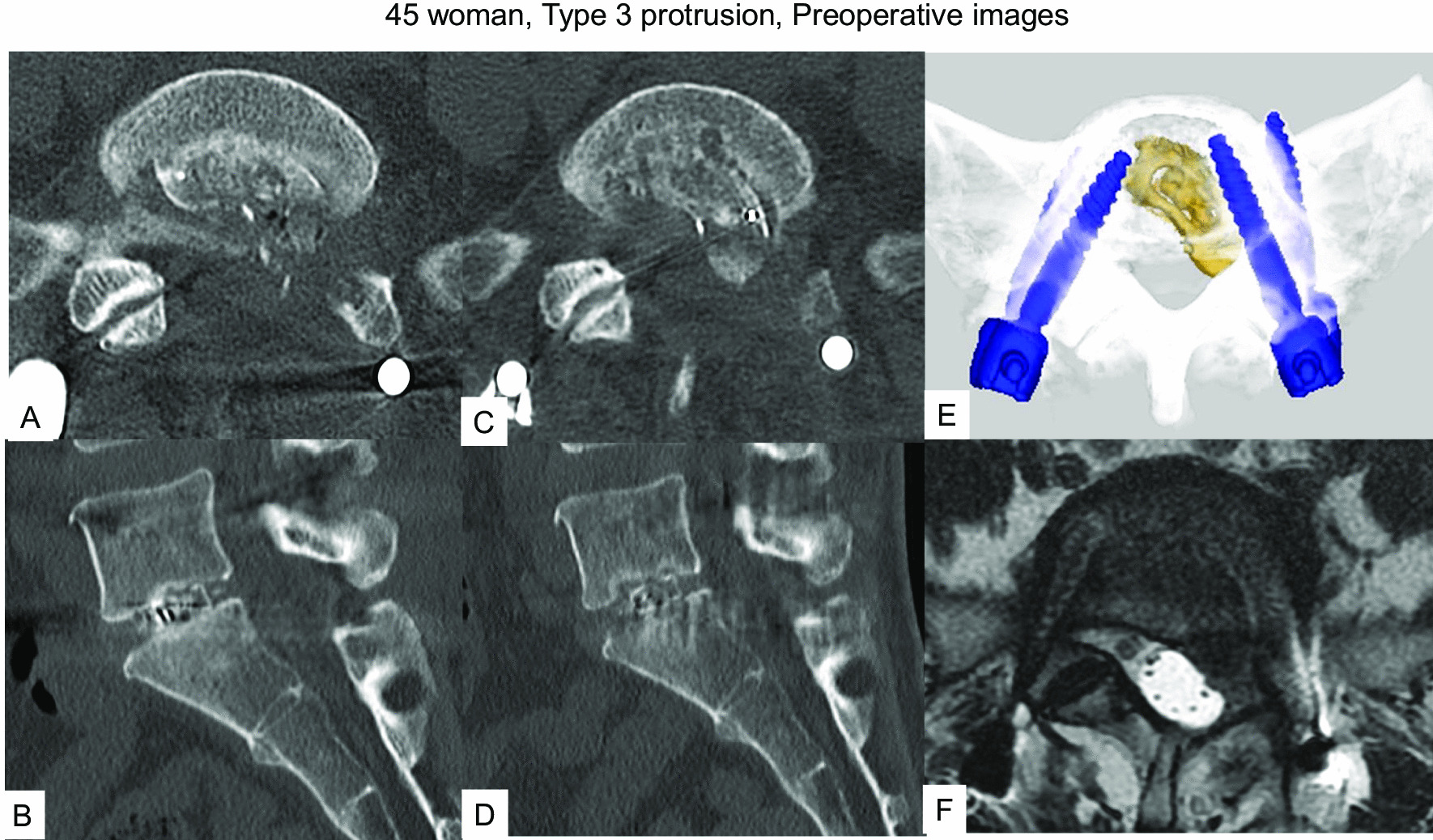
45 woman, Type 3 protrusion, images before revision surgery **A, B** Images at one month follow-up, **A** Axial CT at L5/S1, **B** Sagittal reconstruction CT, **C–F** Images at three months follow-up, **C** Axial CT at L5/S1, **D** Sagittal reconstruction CT, **E** 3-D CT, **F** Axial T2-weighted MR imaging at L5/S1

**Fig. 9:**
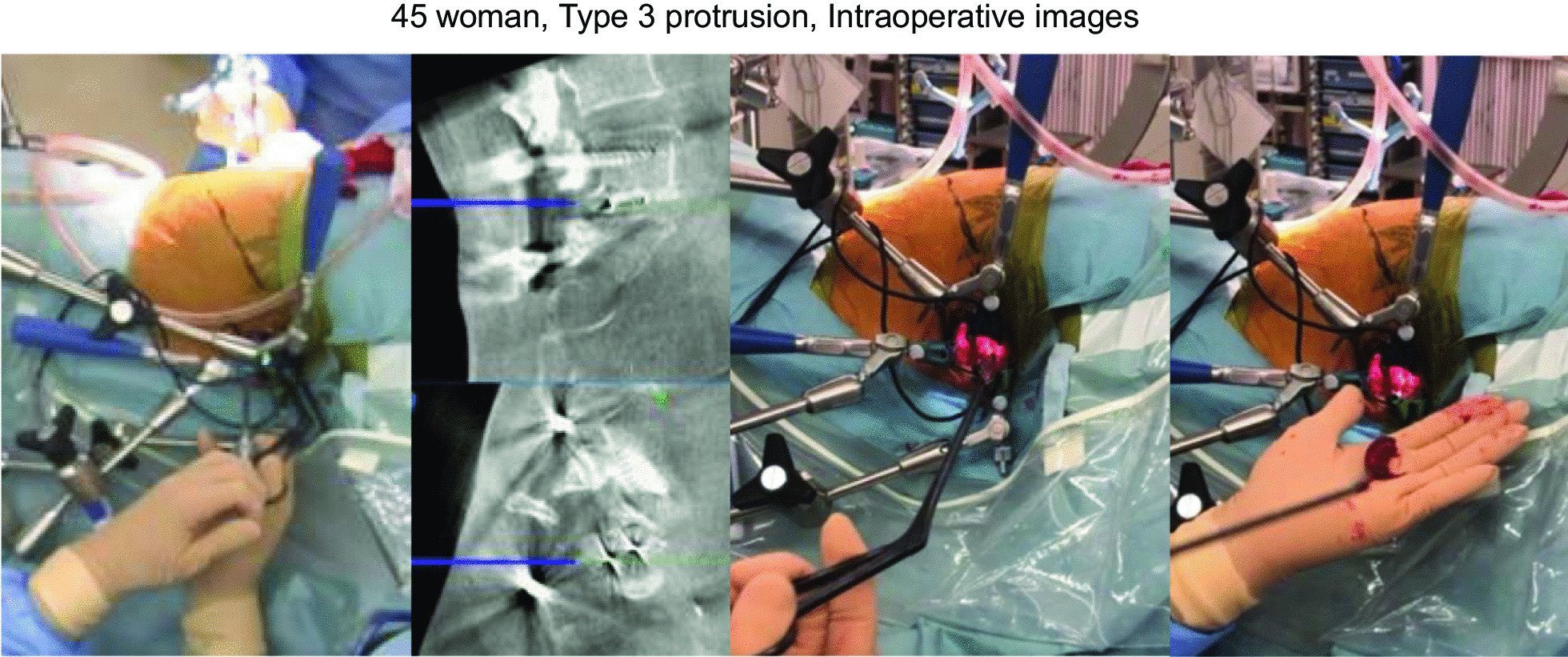
45 woman, Type 3 protrusion, intraoperative images

**Fig. 10:**
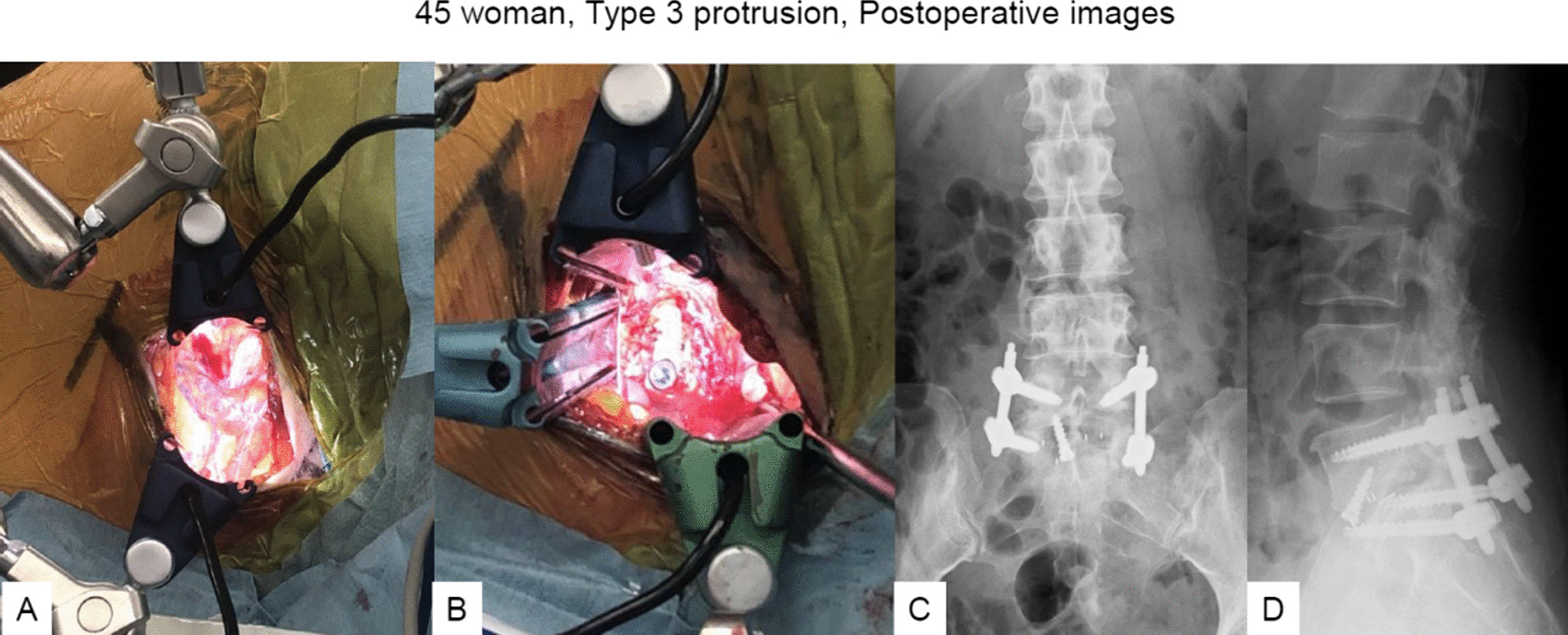
45 woman, Type 3 protrusion, intraoperative and postoperative images **A** Before OLIF51 cage insertion, **B** After OLIF51 cage insertion, **C** Final anteroposterior radiogram, **D** Final lateral radiogram

The patient underwent left L5/S1 TLIF due to re-recurrent lumbar disc herniation (Fig. 8A, B). Her chief complaints were severe left radicular pain, numbness of the S2/3 dermatomes, and urinary disturbance 3 months after TLIF. Follow-up CT and magnetic resonance imaging showed the TLIF cage protruding posteriorly by about one-third of cage diameter, severely compressing the dural sac and S1 nerve root (Fig. 8C–F). After meticulous consideration, posterior removal of the cage was considered likely to prove difficult, so we performed anterior removal of the cage and OLIF51 (Figs. 9, 10).

## Discussion

Lumbar spinal fusion is recognized as an effective treatment for segmental instability. Since Cloward first introduced PLIF more than 70 years ago, this technique has been widely utilized to treat spinal instability and stenosis [1]. TLIF was initially popularized by Harms, as a modification to the PLIF procedure, and gained popularity because of the innovative surgical concept’s ability to eliminate the neural retraction injuries inherent to the traditional PLIF procedure [2]. MI-TLIF has evolved as an ideal treatment strategy for a wide variety of lumbar conditions. Complications commonly associated with MI-PLIF/TLIF include intraoperative neurological injury, interbody implant migration, dural tear, and surgical site infection. Dural tears (frequency, 4.6%) [6, 7], infection (frequency, 2%) [3, 8], screw misplacement (nerve root impingement; frequency, 1–3%) [9], retroperitoneal injury (rare) [10], wrong-level surgery (frequency, 0.03–0.04%) [11] and interbody cage migration (frequency, 2%) [12–14] have all been reported and must be considered in the planning and execution of these surgeries. The incidence of cage migration is reportedly 2.5–6.3% [4, 15, 17]. In this study, the frequency of cage migration was 4.4%. Migration of the intervertebral cage is a relatively rare but potentially serious complication, placing adjacent neural elements at risk, and in some cases heralding nonunion. If a cage migrates forwards into the retroperitoneum or backwards into the vertebral canal, mispositioned cages can have serious clinical consequences. Of these, posterior migration is the more serious due to the risks of nerve root compression or cauda equina syndrome, intensifying neurological symptoms and making the fusion unsuccessful, as described previously [13, 15, 16].

A bullet-shaped cage, higher posterior disc height, presence of scoliotic curvature, and undersized cages have been reported as possible risk factors for cage migration [17]. In our study, risk factors for cage migration were younger age, male sex, and fusion at the L5/S1 level. The mean postoperative duration to onset of cage migration was 3.2 months (range, 2–6 months). Migration has been shown to be connected to small cage size, cage type, inadequate anterior seating of the cage, multilevel fusion procedures and osteoporosis [14, 18]. Surgeon experience less than 3 years and lumbar spondylolisthesis appear to be significantly associated with posterior cage migration [17]. Several studies have emphasized the importance of preserving vertebral bone endplates to prevent cage migration, and techniques to achieve this are very important and in high demand [19, 20]. To minimize the risk of cage migration, we should place the cage with the center located posterior to the disc center. Preparation of the disc and endplate plays an important role in avoiding cage migration. Before cage insertion, a trial cage should reach an adequate depth after disc preparation and bone graft packing. Monitoring of cage position in real time during surgery by fluorescent imaging is also indispensable. The depth ratio on lateral radiography offers a useful tool to detect malpositioned cages. Once decompression and instrumented fusion have been completed, adequate compression must be applied via the pedicle screws to prevent cage migration.

Revision surgeries for cage migration include replacement of the superior graft material, proper end-plate preparation, correction of any technical errors, enhancement of biological fusion, and improvement of the biomechanical environment [21]. However, revision surgery for cage migration is technically demanding. With the posterior approach, dural retraction and nerve root mobilization are difficult because of massive epidural fibrosis, leading to postoperative leg pain or palsy. The safety and efficacy of anterior lumbar interbody fusion (ALIF) as a salvage option for failed posterior lumbar fusion surgery have been demonstrated [22]. In this study, we have proposed a new, treatment-based classification for symptomatic posterior cage migration (Fig. 1). The most common type in our cohort was type 2, involving nerve root compression causing severe radicular pain. However, the most difficult case is type 3, which results in cauda equina syndrome and usually requires cage removal from an anterior approach (Fig. 9). If the only symptom is low back pain due to pseudoarthrosis (type 1), removal of the migrated cage may not even be necessary. Anterior approach or contralateral side approach TLIF is also indicated. For type 2 migrated cage, a removal of the migrated cage is mandatory. In this type, the same side TLIF approach is indicated. For type 3 migrated, a removal of the migrated cage from anterior approach is the best option because posterior approach is a little risky to remove the cage. To the best of our knowledge, no classification of cage migration has previously been reported. This therefore appears to be the first classification of cage protrusion in the literature.

The use of intraoperative navigation has also been shown to provide greater accuracy and less variation in device placement. In this study, we utilized a navigated osteotome, navigated high-speed burr, and the OLIF51 procedure for revision surgeries. To date, few studies have reported the clinical usage of spinal intraoperative O-arm-based navigation with ALIF for cage backout after TLIF/PLIF. Phan et al*.* reported one case in which ALIF and total disc replacement surgery were performed with intraoperative CT-based navigation, showing good results [23]. Park et al. described this method of navigation as safe, feasible and apparently accurate in LLIF procedures [24]. Our previous study showed improvements in the accuracy of screw positioning and a decrease in the misplaced screw rate to 3.7% among scoliosis patients with O-arm navigation [9]. O-arm-navigated surgery has been validated as a surgical intervention for lumbar revision surgery. O-arm navigation techniques offer theoretically sound advantages, appear to represent viable options for salvage operations and are safe in well-trained hands.

This case series yielded a large amount of valuable information regarding cage migration. However, this study did have some limitations that should be kept in mind. First, because of the low incidence, the total number of cage migration cases was insufficient to draw many definitive conclusions. Second, the study was conducted retrospectively without randomization. Third, in addition to cage position and cage height, whether sagittal alignment parameters for the spine represent risk factors have yet to be determined. These parameters were not included in the present study because whole-spine radiographs were not available during the period of case collection. Further studies paying attention to such aspects are needed to clarify which factors influence cage migration.

## Conclusions

Our new classification for cage migration appears useful to create viable strategies for addressing symptomatic lumbar pseudoarthrosis requiring revision surgery due to cage protrusion after MI-PLIF/TLIF. Cage removal with a navigated curette, osteotome, and high-speed burr is an effective technique that reduces surgical time and blood loss. Anterior removal of the cage and OLIF51 should be considered for type 3 protrusion (cauda equine syndrome and/or severe radicular symptom). This new procedure reduces radiation exposure to the surgeon and operating room staff compared with conventional fluoroscopic techniques.

## Data Availability

The datasets used and/or analyzed in the present study are available from the corresponding author upon reasonable request.

## Abbreviations

ALIF: Anterior lumbar interbody fusion
CT: Computed tomography
MRI: Magnetic resonance imaging
MI: Minimally invasive
OLIF: Oblique lumbar intervertebral fusion
PPS: Percutaneous pedicle screw
PLIF: Posterior lumbar interbody fusion
TLIF: Transforaminal lumbar interbody fusion
